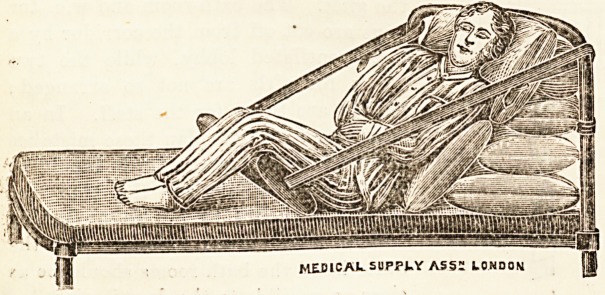# New Appliances & Things Medical

**Published:** 1908-12-12

**Authors:** 


### NEW APPLIANCES & THINGS MEDICAL.

### TWO NEW BISCUITS.

Messrs. Huntley and Palmer, the famous biscuit manufacturers, of Reading, have lately brought out two new varieties of biscuit which deserve the attention of the medical profession. In each the proportion of nitrogenous matter, in the form of protein, is particularly large. Both varieties of biscuit are unsweetened, and the specimens we have received are agreeable in appearance, palatable, and crisp in texture, and in other respects worthy of their manufacturers' reputation.

"*Spartan*" *Biscuits* contain approximately 23 per cent. of protein, and are thus very rich in albumin. They contain about the same percentage of carbohydrates as ordinary bread. The protein is present in a digestible form, and the biscuits are pleasant to the taste and highly nutritious. They should prove of especial value to travellers and athletes, and we might add to those medical men who are engaged in much night-work.

"*Apax*" Biscuits contain an even higher proportion of protein, while the carbohydrate constituents are materially reduced in quantity. The percentages of protein and of carbohydrates are respectively 34 per cent. and 40 per cent. Like the former variety Apax biscuits are unsweetened, and in the same way they are palatable and possess a distinct flavour of their own. Their composition renders them especially suitable to patients with a tendency to corpulency. They are also suitable in those cases of diabetes in which a strict noncarbohydrate diet is not considered desirable, but in which a nutritious and digestible food, relatively poor in starch and sugars, is indicated. For strict diabetic diet the manufacturers, so we understand, prepare a third kind of biscuit called "Akoll" which is stated to be entirely free from carbohydrates. The two varieties we have examined can be recommended with confidence to the profession and the public.

### THE "FORSYTH" SLING-PILLOW.

This "sling-pillow" has been devised for keeping patients in the semi-recumbent and "Fowler" positions. Propping up by means of pillows alone is not sufficient, as the patient slips down from his own weight. Several methods have been tried to overcome this. The above-mentioned "sling-pillow" is one of these. The patient is well propped up by means of pillows or a bed rest. The "sling-pillow" is placed under the thighs, and the straps are fastened to the top bar of the bed-head. In this way the body may be raised at any angle. The "sling-pillow" will be found very useful in the after treatment of abdominal section cases, especially those in which the operation has been in the upper abdomen. It is also useful in nursing cases of pneumonia, bronchitis, and pericarditis. The pillow, which has been devised by Mr. Cairns Forsyth, is made by the Medical Supply Association, 228-230 Gray's Inn Road, London.

## Figures and Tables

**Figure f1:**